# Determination of Suitable RT-qPCR Reference Genes for Studies of Gene Functions in *Laodelphax striatellus* (Fallén)

**DOI:** 10.3390/genes10110887

**Published:** 2019-11-04

**Authors:** Wei Wu, Haoqiu Liu, Yan Dong, Yun Zhang, Sek-Man Wong, Changchun Wang, Yijun Zhou, Qiufang Xu

**Affiliations:** 1 Institute of Plant Protection, Jiangsu Academy of Agricultural Sciences, Key Laboratory of Food Quality and Safety of Jiangsu Province, State Key Laboratory Breeding Base, Nanjing 210014, China; ztaiww1314@163.com (W.W.); liuhaoqiu@u.nus.edu (H.L.); dongy19870330@163.com (Y.D.); zhangyunzjnu@163.com (Y.Z.); 2 National University of Singapore (Suzhou) Research Institute, Department of Biological Sciences, National University of Singapore, Kent Ridge 117543, Singapore; dbswsm@nus.edu.sg; 3 College of Chemistry and life Sciences, Zhejiang Normal University, Jinhua 32100, China; wcc@zjnu.cn

**Keywords:** *Laodelphax striatellus*, RT-qPCR, reference gene, rice black-streaked dwarf virus, rice stripe virus

## Abstract

The reverse transcription quantitative polymerase chain reaction (RT-qPCR) has been widely used to determine gene functions in *Laodelphax striatellus* (Fallén) (small brown planthopper). Selection of suitable reference gene(s) for normalizations of RT-qPCR data is critical for reliable results. To date, reports on identification of suitable *L. striatellus* reference genes are still very limited. *L. striatellus* is a destructive rice pest and it can transmit multiple viruses, including *Rice black-streaked dwarf virus* (RBSDV), *Rice stripe virus* (RSV), and *Maize rough dwarf virus* (MRDV), to many important cereal crops worldwide. In this study, we examined the stablity of seven selected candidate reference genes in *L. striatellus* at different developmental stages, in different tissues, in RBSDV- or RSV-infected *L. striatellus* or in RBSDV-infected and *Lssynaptojanin 1* (*LsSYNJ1*)-silenced *L. striatellus*. The RT-qPCR data representing individual candidate genes were analyzed using five different methods: the delta Ct method, geNorm, NormFinder, BestKeeper, and the RefFinder algorithm, respectively. The most stable reference gene for the specific condition was selected according to a comprehensive analysis using the RefFinder method. Ribosomal protein L5 (*LsRPL5*) and *LsRPL8* are the most stably expressed genes in *L. striatellus* at different developmental stages. Alpha-1-tubulin (*Lsα-TUB*) is the most stably expressed reference gene in different tissues of RBSDV viruliferous (RBSDV-V) or non-viruliferous (RBSDV-NV) *L. striatellus*. *LsRPL8* is the most stably expressed reference gene in RBSDV-V or RSV viruliferous (RSV-V) *L. striatellus*, while beta-tubulin (*Lsβ-TUB*) is the most stably expressed reference gene in RBSDV-V and *LsSYNJ1*-silenced *L. striatellus*. The selected reference genes were further investigated during analyses of RBSDV *P5-1* and *P10* gene expression in different tissues from RBSDV-V or RBSDV-NV *L. striatellus*. The stably expressed reference genes identified in this study will benefit future gene function studies using *L. striatellus*.

## 1. Introduction

*Laodelphax striatellus* (Fallén) (small brown planthopper), Order: Hemiptera, Family: Delphacidae, is an important agricultural pest which attacks plants by sucking sap from pholem with its stylet. *L. striatellus* is also an important insect vector for many plant viruses, including *Rice black-streaked dwarf virus* (RBSDV), *Rice stripe virus* (RSV), and *Maize rough dwarf virus* (MRDV) [1,2,3]. Among these viruses, RBSDV and RSV are known to cause devastating damages to multiple important cereal crops in many countries [3,4,5]. Studies have shown changes in insect gene, protein, and microRNA expression upon virus infection, and accumulation of virus-derived siRNAs in *L. striatellus* through various technologies [6,7,8,9,10,11]. These published data have provided us with comprehensive information on gene expression in *L. striatellus* upon virus infection, and on the interactions between virus and host genes in *L. striatellus*.

The reverse transcription quantitative polymerase chain reaction (RT-qPCR) is a sensitive and quantitative assay for determinations of RNA transcript levels in plants, animals, and microorganisms [12,13,14]. RT-qPCR has been used to investigate gene expression in different tissues of *L. striatellus* nymphs, and in *L. striatellus* infected with different viruses [15,16]. It has also been used to validate the expression of specific genes identified through various transcriptomic and proteomic approaches, specific RNA transcript levels after the gene was silenced in *L. striatellus*, and virus accumulation in infected *L. striatellus* [6,7,15,17]. Because the reliability of RT-qPCR depends largely on the reference gene(s) used to normalize the data, it is crucial to know the stability of the reference gene(s) under specific experimental conditions prior to RT-qPCR [18]. Several *L. striatellus* reference genes have been investigated for their expression in *L. striatellus* nymphs at different instar stages before the release of *L. striatellus* genome sequences [16]. However, analyses of *L. striatellus* reference gene expression in *L. striatellus* under other experimental conditions are still lacking.

Previous studies have selected some housekeeping genes as reference genes to normalize RT-qPCR data in *L. striatellus*. For example, the expression of the RSV nucleocapsid protein gene (*CP*) in *L. striatellus* was determined after normalization of the data against the expression level of reference gene *actin* [19]. The *Actin* gene expression was also used as an internal control during RT-qPCR to investigate host gene expression patterns or to verify the RNA levels of differentially expressed genes in *L. striatellus* upon virus infection [20,21]. The 18S ribosome RNA (*18S rRNA*) gene was used as an reference gene for the quantification of RSV accumulation in *L. striatellus* at various developmental stages [22]. In a different report, ADP ribosylation factor (*LsARF*) was used to normalize the expression data of nuclear receptor *E75* (*LsE75*), which is known to suppress RSV transmission through *L. striatellus* [17]. The *L. striatellus* elongation factor 2 (*LsEF2*) was used as a reference gene to determine the expression of host and viral genes in *L. striatellus* [23]. However, the expression stabilities of these reference genes under the specific experimental conditions were not mentioned.

Several research laboratories have investigated the reliabilities of reference genes in other planthoppers in the Family Delphacidae for RT-qPCR. For example, ribosomal protein S15e *(RPS15)* and ribosomal protein S11 (*RPS11*) were reported to be the most suitable reference genes in different *Nilaparvata lugens* (brown planthopper) tissues and at different developmental stages [24]. Glyceraldehyde-3-phosphate dehydrogenase (*GAPDH*) was reported as the most suitable reference gene for *Southern rice black-streaked dwarf virus* (SRBSDV)-infected *Sogatella furcifera* (white-backed planthopper), and *RPL9* as the most suitable reference gene for different *S. furcifera* tissues [25]. For *Mal de Río Cuarto virus* (MRCV)-infected *Delphacodes kuscheli* (planthopper), polyubiquitin C (*UBI*), ribosomal protein S18 (*RPS18*) and actin (*ACT*) were found to be the most suitable RT-qPCR reference genes [26]. Because a suitable reference gene in one insect may not be the same as in other insects, or in the same insect under different experimental conditions, the suitability of individual housekeeping genes for RT-qPCR data normalization must be checked under specific conditions prior to use.

In this study, we selected seven commonly used housekeeping genes with different expression levels for a systematic analysis. We evaluated *LsACT*, *Lsα-TUB*, *Lsβ-TUB*, *LsGAPDH*, *LsRPL5*, *LsRPL8*, and *Ls18S rRNA* for their usefulness as reference genes through RT-qPCR using *L. striatellus* at different developmental stages, infected or not infected with a virus, in different *L. striatellus* tissues, and in RBSDV-infected and *LsSYNJ1*-silenced *L. striatellus*. Five statistical algorithms: the delta Ct method, geNorm, NormFinder, Best-Keeper, and RefFinder, were employed to determine the expression stabilities of these candidate reference genes. The selected suitable reference genes described here should benefit gene function studies in *L.striatellus*.

## 2. Materials and Methods

### 2.1. Insect Rearing and Virus Acquisition

Non-viruliferous (NV) and RSV viruliferous *L. striatellus* was originally collected from Haian, in the Jiangsu Province, China, and maintained in the laboratory as described previously [2]. The insects were reared on rice seedlings inside a chamber set at 25 ± 3 °C, with a 16:8 h (light:dark) photoperiod, and 55 ± 5% relative humidity. The RBSDV viruliferous (RBSDV-V) insects were obtained by allowing second instar nymphs to feed on RBSDV-infected rice seedlings for two days to acquire the virus, and then to feed on healthy rice seedlings for another two days.

### 2.2. Preparation of Insect Samples for RT-qPCR

(1) *L. striatellus* at different developmental stages: Embryos (24 h post laying), second instar nymphs, fifth instar nymphs, and adult *L. striatellus* were collected. Approximately 50 individual insects at a specific developmental stage were collected for the assay and each treatment included three biological replicates.
(2) Different insect tissues: Head, cuticle, midgut, and fat body from RBSDV-V and RBSDV-NV third instar *L. striatellus* were collected using a fine-pointed tweezer (Dumont, Switzerland). Insects were immerged in the 1× PBS (pH 7.2), and tissues were collected under stereo microscope. RBSDV-V *L. striatellus* used for tissue collection was prepared by allowing second instar *L. striatellus* nymphs to feed on RBSDV-infected rice seedlings for 7 days, and then on healthy rice seedlings for 7 days. This experiment was repeated three times and in each experiment around 200 insects were used.
(3) RBSDV-V *L. striatellus* was prepared by allowing second instar *L. striatellus* nymphs to feed on RBSDV-infected rice seedlings for 2 days. The insects were then allowed to feed on healthy rice seedlings for 7, 14, and 21 days, respectively. Insects at the same developmental stage reared on healthy rice seedlings were used as controls. Progenies of RSV-V or RSV-NV *L. striatellus* were collected and used as the RSV-V or RSV-NV samples for the assays. The presence of RBSDV or RSV in viruliferous *L. striatellus* was confirmed by RT-PCR using RBSDV *P10-* or RSV *CP*-specific primers prior to sampling (Table S1). For each treatment, three groups with 50 insects each were used.
(4) Lssynaptojanin 1 (*LsSYNJ1*)-silenced and RBSDV-V *L. striatellus* was prepared for reference gene analysis, and the Lssynaptojanin 1 (*LsSYNJ1*)-silenced insect was used as control. A fragment (654 bp) representing a partial sequence of *LsSYNJ1* was RT-PCR amplified using specific primers (Table S1). A similar-sized fragment representing a partial sequence of *GFP* gene was also amplified. The two fragments were cloned individually into the pMD18-T vector (TaKaRa, Dalian, China) and sequenced. dsRNA from the two vectors was synthesized using the Transcript Aid T7 High Yield Transcription kit (Thermo Scientific, Waltham, MA, USA). The resulting dsRNA was injected into third instar nymphs (100 ng per nymph) using a FemtoJet express instrument (Eppendorf, Hamburg, Germany). Efficiency of *LsSYNJ1* silencing was determined by RT-qPCR at 3 days post injection. After analysing the knockdown efficiency of *LsSYNJ1*, the dsRNA-injected insects were divided into two groups. One group fed on RBSDV-infected rice seedlings for 2 days to acquire virus and then fed on healthy rice seedlings for another 2 days. Another group was grown on healthy rice seedlings for 4 days, and then used for analyses of reference gene expression. Approximately 30 insects were collected as a group, and three replications were applied for each treatment. The experiment was repeated three times.

### 2.3. Total RNA Extraction and cDNA Synthesis

Insect samples were stored inside 1.5 mL RNase-free microfuge tubes at −80 °C until use. Total RNA was isolated from individual samples using the RNAiso plus reagent (Takara, Dalian, China). The quality and quantity of isolated RNA samples were individually analyzed using a NanoDrop2000 (Thermo Scientific, Waltham, MA, USA). RNA samples with the 260/280 ratio between 1.80 and 2.0, and the 260/230 ratio between 2.00 and 2.20 were used for RT-qPCR. Before reverse transcription, each total RNA sample was checked again through electrophoresis in 1% agarose gels. Reverse transcription was performed using the PrimeScript^TM^ RT Reagent Kit supplemented with a gDNA Eraser (Takara, Dalian, China). Here, 1 μg total RNA was used in each 20 μL reaction mixture, according to the manufacturer’s protocol.

### 2.4. Primer Design

Seven *L. striatellus* genes: *LsACT*, *Lsα-TUB*, *Lsβ-TUB*, *LsGAPDH*, *LsRPL5*, *LsRPL8*, and *Ls18S rRNA* were selected as candidate reference genes. Sequences of these genes were retrieved from the GenBank. The accession numbers and primer sequences of the genes are shown in Table 1. Primers specific to each gene were designed individually using the Primer Premier 5.0 software (PREMIER Biosoft, Davis, CA, USA). The size and sequence of each PCR product were checked through 1% agarose gel electrophoresis and DNA sequencing.

### 2.5. Quantitative PCR

Quantitative PCR reactions were conducted on a Bio-Rad iQ5 Real-time PCR system. Each reaction was performed in a 20-μL volume with 1 μL cDNA (10 ng), 8 μL ddH_2_O, 0.5 μL of each primer, and 10 μL Power SYBR Green PCR Master Mix (Takara, Dalian, China). The PCR condition was 95 °C for 5 min, followed by 40 cycles at 95 °C for 10 s and 60 °C for 30 s. The melting curves and standard curves were analyzed to ensure specificity of the amplified products.

### 2.6. Analyses of Candidate Gene Expression

Five different statistical algorithms were used to evaluate the stabilities of candidate reference gene expression. The geNorm algorithm was used to calculate the gene expression stability by measuring the M values, and to determine the optimal number of reference genes for this study as described previously [27]. The NormFinder algorithm utilized a linear mixed effect model to estimate both intra- and inter-group variations, and to rank the candidate reference genes by the stabilityvalues (SV) [28]. The BestKeeper algorithm was used to evaluate the expression stability of individual candidate reference genes based on the standard deviations (SD) and the coefficients of variation (CV) [29]. The RefFinder algorithm is a web-based analysis tool (https://omictools.com/reffinder-tool), which integrates the results obtained using the geNorm, Bestkeeper, Normfinder, and delta Ct method, and then ranks the candidate reference genes based on the geometric mean values (GM) [30].

### 2.7. Validation of Candidate Gene Expression

To validate the expression stability of individual selected reference genes, RT-qPCR assays were conducted using the cDNA samples from different tissues and *LsSYNJ1*-silenced samples of RBSDV-V *L. striatellus*. We quantified the expression levels of RBSDV *P10* and *P5-1* in different RBSDV-V *L. striatellus* tissues, including head, cuticle, fat body, and midgut, through RT-qPCR. The results were normalized using the expression level of *Lsα-TUB* (the most stably expressed reference gene) or *LsRPL8* (the least stably expressed reference gene) with the RefFinder method. The insects used in this study were reared on healthy rice seedlings for 7 days after two-day acquisition on RBSDV-infected rice seedlings. In *LsSYNJ1*-silenced samples, the best reference gene *Lsβ-TUB* and the worst reference gene *LsGAPDH* were used for RT-qPCR data normalization. Relative expression of RBSDV *P5-1* and *P10* were calculated using the 2 ^−^^△△Ct^ method. Primers for RBSDV *P5-1* and *P10* are described in Table S1. Statistical analysis was performed by the Student’s *t*-test based on the independent sample *t*-test.

## 3. Results

### 3.1. Expression Profiles of Candidate Reference Genes

**Figure 1:**
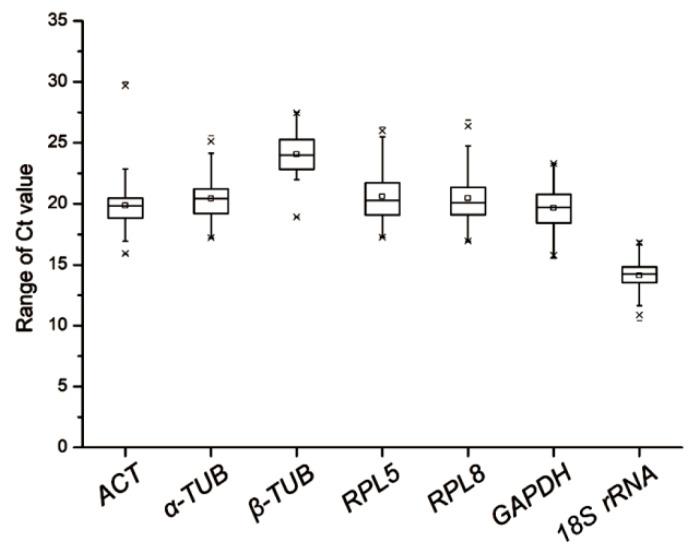
Ranges of expression of the seven selected reference genes in all analyzed samples. The whisker box plots represent the cycle threshold numbers (Ct value) of the seven reference genes. The horizontal lines inside the boxes are the median values, and the whiskers of the boxes are the minimum and maximum Ct values.

Relative expression of the seven selected candidate reference genes in *L. striatellus* at various developmental stages, in different tissues of RBSDV-V or RBSDV-NV *L. striatellus*, in RBSDV-V or RSV-V *L. striatellus*, and in RBSDV-V and *LsSYNJ1*-silenced *L. striatellus*, were determined through RT-qPCR. Analyses of melting curves and agarose gel electrophoresis results showed that the PCR primers used in this study were gene-specific (Figures S1 and S2). When the results from all the test samples were summarized and compared, we found that the calculated Ct values of these seven candidate genes varied from 14.10 to 24.07. According to these Ct values, *Ls18S rRNA* showed the highest expression level, while *Lsβ-TUB* showed the lowest (Figure 1 and Table 2). Based on the standard deviation (SD) values, *Ls18S rRNA* showed the lowest expression variations among different samples (mean Ct value ± SD = 14.10 ± 1.22). *LsRPL5* and *LsRPL8* showed the highest expression variations among the seven genes (i.e., 20.61 ± 2.10 and 20.46 ± 2.22, respectively).

### 3.2. Expression of Candidate Genes in L. striatellus at Different Developmental Stages

**Figure 2:**
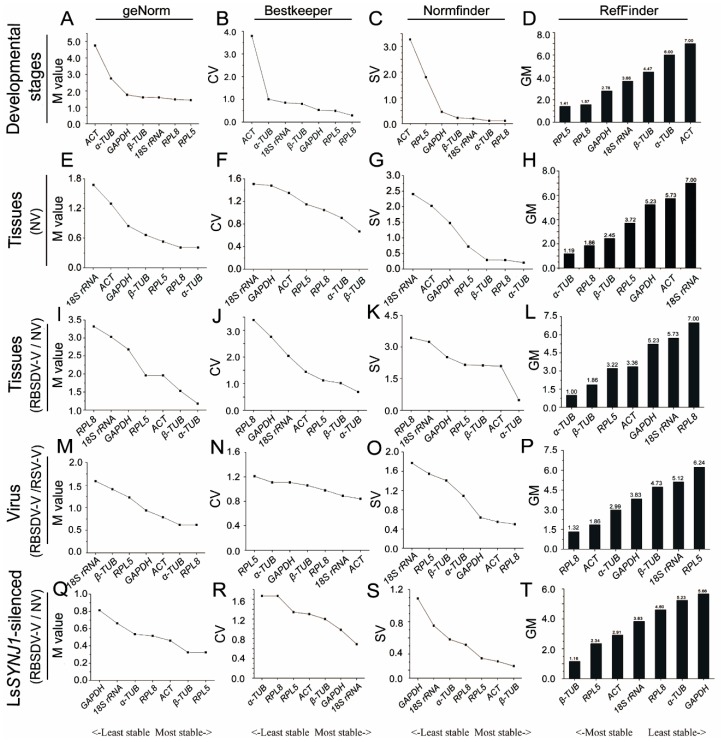
Rankings of reference gene expression stabilities determined by RT-qPCR followed by geNorm, Bestkeeper, Normfinder, or the RefFinder algorithm. Results represent individual reference genes. (**A**–**D**) *L. striatellus* at all developmental stages; (**E**–**H**) tissues from non-viruliferous (NV) *L. striatellus*; (**I**–**L**) tissues from *Rice black-streaked dwarf virus* viruliferous (RBSDV-V) or NV *L. striatellus*; (**M**–**P**) RBSDV-V or *Rice stripe virus* viruliferous (RSV-V) *L. striatellus*; (**Q**–**T**): RBSDV-V and Lssynaptojanin 1 (*LsSYNJ1*) -silenced *L. striatellus*, or RBSDV-NV and *LsSYNJ1*-silenced *L. striatellus*.

The expression levels of the seven candidate genes in *L. striatellus* at different developmental stages were first determined by RT-qPCR and then analyzed for their expression stabilities by the four statistical algorithms (Table S2). The results obtained using the geNorm algorithm showed that *LsRPL5* and *LsRPL8* were the most stably expressed genes in *L. striatellus* at different developmental stages (Figure 2A). Analyses of the RT-qPCR data using the Bestkeeper or Normfinder algorithm also showed that *LsRPL8* and *LsRPL5* were the most stably expressed genes in *L. striatellus* at different developmental stages (Figure 2B,C). Analysis of the RT-qPCR data using the RefFinder algorithm showed that *LsRPL5* was the most stably expressed genes in *L. striatellus* at different developmental stages. The stability ranking of the other candidate genes from the more stable to the least stable was *LsRPL8*, *LsGAPDH*, *Ls18S rRNA*, *Lsβ-TUB*, *Lsα-TUB* and *LsACT* (Figure 2D).

### 3.3. Expression of the Candidate Genes in Different L. striatellus Tissues

To determine the expression of these candidate genes in different *L. striatellus* tissues, we analyzed their expression in *L. striatellus* head, cuticle, midgut, and fat body obtained from RBSDV-V and RBSDV-NV *L. striatellus* through RT-qPCR. The presence of RBSDV in different *L. striatellus* tissues was first confirmed by RT-PCR using RBSDV *P10*-specific primers prior to the assay (Figure S3). Analyses of the RT-qPCR data using the geNorm or Normfinder algorithm showed that *Lsα-TUB* was the most stably expressed candidate gene. Analysis of the RT-qPCR data using the Bestkeeper algorithm showed that *Lsβ-TUB* was the most stably expressed gene in various tissues from RBSDV-NV *L. striatellus*. Based on the RefFinder algorithm, *Lsα-TUB* was the most stably expressed gene in various tissues of RBSDV-NV *L. striatellus* (Figure 2E–H).

When the RT-qPCR data from different tissues of RBSDV-V or RBSDV-NV *L. striatellus* were compared, *Lsα-TUB*, *Lsβ-TUB,* and *LsRPL5* were the most stably expressed genes, using the Bestkeeper or geNorm algorithm. When the Normfinder algorithm was used to analyze the same RT-qPCR data, *Lsα-TUB*, *LsACT*, and *Lsβ-TUB* were the most stably expressed genes. Analysis using the RefFinder algorithm showed that *Lsα-TUB*, *Lsβ-TUB,* and *LsRPL5* were the most stably expressed genes (Figure 2I–L).

### 3.4. Expression of the Candidate Genes in RBSDV-V or RSV-V L. striatellus

RBSDV-V or RSV-V *L. striatellus* was collected and utilized to determine the expression of the candidate genes through RT-qPCR. The presence of RBSDV or RSV in *L. striatellus* was first confirmed by RT-PCR using RBSDV *P10* or RSV *CP* gene-specific primers prior to the assays (Figure S3). Analyses of the RT-qPCR data using the geNorm or Normfinder algorithm showed that *LsRPL8* was the most stably expressed gene in virus-infected *L. striatellus.* However, when the Bestkeeper algorithm was used to analyze the RT-qPCR data, *LsACT* became the most stably expressed gene (Figure 2M–O). Analysis of RT-qPCR data using the RefFinder algorithm showed that *LsRPL8, LsACT*, and *Lsα-TUB* were the most stably expressed genes, followed by *LsGAPDH* and *Lsβ-TUB* (Figure 2P).

### 3.5. Expression of the Candidate Genes in RBSDV-V and LsSYNJ1-Silenced L. striatellus

RNA interference has now been widely used to study gene functions during virus transmission through their insect vectors [15,17,19]. To determine whether RNA interference can affect the expression of reference genes, we first silenced *LsSYNJ1* expression in *L. striatellus* through microinjection. RT-qPCR analysis showed that *LsSYNJ1* expression was knockdown at three days after microinjection (Figure S4). The *LsSYNJ1*-silenced insects were then divided into two groups and fed on healthy and RBSDV-infected rice seedlings, respectively.

Expression of the candidate genes in the *LsSYNJ1*-silenced *L. striatellus* fed on healthy and RBSDV-infected rice seedlings was determined by RT-qPCR (Figure S1). Analysis of the RT-qPCR data using the geNorm algorithm showed that *LsRPL5*, *Lsβ-TUB*, and *LsACT* were the most stably expressed genes. Analysis of the RT-qPCR data using the Normfinder algorithm ranked *Lsβ-TUB*, *LsACT*, and *LsRPL5* as the most stably expressed genes. When the Bestkeeper algorithm was used to analyze the RT-qPCR data, *Ls18S rRNA*, *LsGAPDH,* and *Lsβ-TUB* were the most stably expressed genes. Analysis of the RT-qPCR data using the RefFinder algorithm showed that, for RBSDV-V and *LsSYNJ1*-silenced *L. striatellus*, *Lsβ-TUB* was the most suitable reference gene (Figure 2Q–T).

### 3.6. Overall Ranking of the Candidate Genes

Besides analyzing the best reference genes for individual treatments, an overall ranking of the candidate genes were also analyzed in *L. striatellus* at different developmental stages, in different *L. striatellus* tissues, in RBSDV-V or RSV-V *L. striatellus*, and in RBSDV-V and *LsSYNJ1*-silenced *L. striatellus* using the four different algorithms. Analysis using the Bestkeeper algorithm showed that *Ls18S rRNA* was the best reference gene, while the analysis using the geNorm or Normfinder algorithm showed that *Lsα-TUB* was the most suitable reference gene. The comprehensive ranking generated by the RefFinder algorithm showed that *Lsα-TUB* was the best RT-qPCR reference gene for *L. striatellus*. The stability ranking of the other candidate genes was *Lsβ-TUB* > *LsGAPDH* > *Ls18S rRNA* > *LsRPL5* > *LsACT* > *LsRPL8*, from the more stable to the least stable (Table 2).

### 3.7. Determination of the Optimal Number of Reference Genes Needed for RT-qPCR Normalization

**Figure 3:**
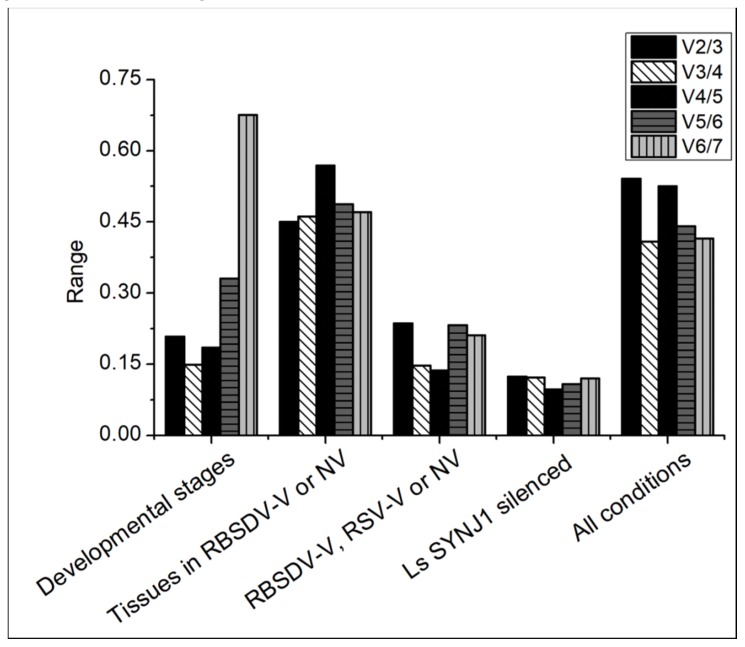
Pairwise variations were calculated using the geNorm algorithm to determine the optimal numbers of reference genes needed for accurate RT-qPCR data normalization. The pairwise variation (V_n_/V_n+1_) was calculated using data from different developmental stages, different tissues, RBSDV-NV, RBSDV-V, or RSV-V *L. striatellus*, *LsSYNJ1*-silenced *L. striatellus*, and *L. striatellus* in all tested samples.

To determine whether two or more *L. striatellus* reference genes are needed for RT-qPCR data normalization, pairwise variations were calculated using the geNorm algorithm. The pairwise variation value (V_n_/V_n+1_) below 0.15 indicates that the optimum number of reference gene for RT-qPCR data normalization is n. The V3/4 value shows that three reference genes (i.e., *LsRPL5*, *LsRPL8*, and *LsGAPDH*) are needed to normalize the RT-qPCR data generated from RBSDV-NV *L. striatellus* at different developmental stages. When the V3/4 value from RBSDV-V/RSV-V *L. striatellus* is found to be below 0.15, it indicates that the optimum number of reference genes needed for normalization is three (i.e., *Lsα-TUB*, *Lsβ-TUB*, and *LsRPL5*). When the V2/3 value from RBSDV-NV, RBSDV-V and *LsSYNJ1*-silenced *L. striatellus* is at 0.12, it indicates that two reference genes (i.e., *Lsβ-TUB* and *LsRPL5*) are needed for RT-qPCR data normalization. The geNorm pairwise variation analysis shows that the pairwise variation values from different tissues in RBSDV-V or NV and all the tested combinations were above the threshold value, suggesting that no suitable combinations of reference genes can be used (Figure 3).

### 3.8. Normalization of RT-qPCR Data From Different L. striatellus Tissues Using Reference Gene *Lsα-TUB* and *LsRPL8*

**Figure 4:**
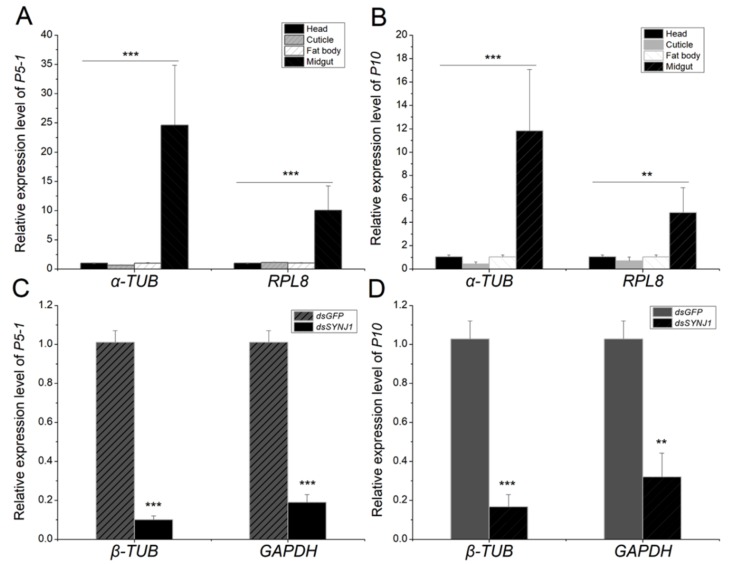
Determinations of RBSDV *P5-1* and RBSDV *P10* expression using different reference genes. (**A**,**B**) The RT-q PCR data were normalized using most suitable reference gene *Lsα-TUB* or the least stable reference gene *LsRPL8* in different tissues including the head (black), cuticle (light gray), fat body (white), and midgut (gray). (**C**,**D**) The RT-q PCR data were normalized using most suitable reference gene *Lsβ-TUB* and least stable reference gene *LsGAPDH* in *dsGFP* (gray) and *LsSYNJ1*-silenced samples (black). **means *p* < 0.005, *** means *p* < 0.001.

To validate the usefulness of the selected reference gene *Lsα-TUB*, we determined the expression levels of RBSDV *P10* and *P5-1* in different tissues and in *LsSYNJ1* silenced from RBSDV-V *L. striatellus* using RT-qPCR. The RT-qPCR data was normalized using the best reference gene *Lsα-TUB* and the worst reference gene *LsRPL8* in different tissues identified through above experiments, and the relative expression was calculated by the 2^-^^△△Ct^ method. The results showed that the expression levels of RBSDV *P10* and *P5-1* in the *L. striatellus* midgut were significantly higher than that in the head, cuticle, or fat body (Figure 4A,B). Using *Lsα-TUB* as the reference gene, the expression of RBSDV *P10* in the midgut was 11.82-fold higher than that in the head. When *LsRPL8* was used as the reference gene, the expression of RBSDV *P10* in the midgut was only 4.82-fold higher than that in the head. Similar results were obtained for RBSDV *P5-1* when the data was normalized against *Lsα-TUB* and compared with the data normalized against *LsRPL8*. When the RT-qPCR data were normalized using the best reference gene *Lsβ-TUB* and the worst reference gene *LsGAPDH* in *LsSYNJ1*-silenced and RBSDV-V samples, similar results were obtained for RBSDV *P10* and RBSDV *P5-1*. The expression of virus gene was much lower in dsRNA injection treatment when using the most suitable reference gene (Figure 4C,D).

## 4. Discussion

Accurate RT-qPCR results depend on several key factors, including data normalization using reliable reference gene(s). Although RT-qPCR has been used to study gene expression and functions in *L. striatellus* in many laboratories [2,6,15,31,32], the expression of the reference genes used in these studies was not characterized in different *L. striatellus* tissues or in *L. striatellus* under various conditions. Because the same reference genes in different insect species or in the same insect species but under different growth or environmental conditions can vary significantly, we consider that the suitability of the reported *L. striatellus* reference genes should be re-evaluated under different defined experimental conditions.

Housekeeping genes, such as *18S rRNA, TUB,* and *ACT*, are often used as the internal control genes to normalize RT-qPCR data. For example, *18S rRNA* is known as a highly expressed cytosolic small ribosomal subunit gene and is conserved among many different insect species [33]. In this study, we found that *18S RNA* was the most abundant gene, compared with the other six candidate genes tested in this study, according to the CT values. However, this gene was not stably expressed in different tissues from RBSDV-NV or RBSDV-V *L. striatellus*. Also, its expression in *L. striatellus* at different developmental stages or in *LsSYNJ1*-silenced *L. striatellus* was only intermediately stable (Figure 2). These findings indicate that *Ls18S rRNA* is not a suitable reference gene for RT-qPCR assays for *L. striatellus* under the conditions described in this paper. In several previous reports, *Ls18S rRNA* was also not considered as the best RT-qPCR reference gene for assays using different tissues from two planthoppers (*S. furcifera and N. lugens*), and these two insects at different developmental stages or after virus infection [24,25]. However, *Ls18S rRNA* was reported as an ideal RT-qPCR reference gene for MRCV-infected planthopper (*D. kuscheli*). Therefore, we propose that whether a housekeeping gene is suitable to serve as a RT-qPCR reference gene needs careful evaluation under the defined experimental conditions.

Tubulin is the basic structural unit of microtubules and functions in many essential cellular processes, such as maintaining cytoskeletal structure [34]. As in an earlier report using SRBSDV-infected *S. furcifera* [25], we ranked *Lsα-TUB* as the most reliable RT-qPCR reference gene for different *L. striatellus* in tissues. The expression of *α-TUB* was reported to be unstable in MRCV-infected *D. kuscheli* [26], but it is relatively stable in *N. lugens* at different developmental stages or in different tissues of the insect [24]. He and colleagues reported earlier that three *TUB* genes were not the most suitable RT-qPCR reference genes for *L. striatellus* at different nymph instar stages [16]. In this study, we found that the expression of *Lsα-TUB* is stable in RBSDV-V and *LsSYNJ1*-silenced *L. striatellus*, but it is not suitable as a RT-qPCR reference gene for *L. striatellus* at different developmental stages. This gene is also not suitable as a RT-qPCR reference gene for virus-infected *L. striatellus* (Figure 2). In summary, because the expression of *α-TUB* varied among different planthopper species, even under the similar experimental conditions, utilization of this gene as a RT-qPCR reference gene should be done with cautioun.

*LsACT* can interact with RBSDV P10 directly [35]. In this study, *LsACT* was ranked as the second most stably expressed reference gene in RBSDV-V or RSV-V *L. striatellus* (Figure 2P). These results suggest that the expression of *LsACT* is not affected significantly by virus infection in *L. striatellus*. However, the expression of *LsACT* in *L. striatellus* at different developmental stages or in different *L. striatellus* tissues varied (Figure 2D,H). Our finding is in accordance with a previous report that expression of *LsACT* is variable in different instar *L. striatellus* nymphs [16]. In addition, the expression of *ACT* in *S. furcifera* at different developmental stages, in different *S. furcifera* tissues, in SRBSDV-V *S. furcifera*, or in *S. furcifera* after different temperature treatments is less stable than several other reference genes analyzed in the same study [25]. In *N. lugens*, the expression of *ACT* is also variable at different developmental stages or in different tissues [24].

The ribosomal proteins are essential for protein synthesis and are involved in multiple cell processes, including cell development, gene transcriptional regulation, cellular proliferation and differentiation [36,37,38]. Although LsRPL5 and LsRPL8 were shown to interact directly with RSV [39], their expression was found to be stable in our study. In fact, *LsRPL5* was found to be the most stably expressed reference gene in *L. striatellus* at different developmental stages, and the second most stably expressed reference gene in virus-infected *L. striatellus* tissues as well as in *LsSYNJ1*-silenced *L. striatellus*. Similarly, *LsRPL8* was found to express stably in virus infected *L. striatellus*, in *L. striatellus* at different developmental stages, or in different *L. striatellus* tissues. Several other planthopper ribosomal protein genes have also been reported as suitable RT-qPCR reference for data normalization. For example, *PRS15* was reported as the most suitable RT-qPCR reference gene for *N. lugens* at different developmental stages [24], while *RPL9* was reported to be stably expressed in different tissues of *S. furcifera* and *N. lugens* [25,40]. Consequently, we consider that many ribosomal protein family genes are likely to be expressed stably in planthopper at different developmental stages or in different tissues.

In summary, we analyzed the expression stabilities of seven selected candidate *L. striatellus* reference genes through RT-qPCR, followed by analyses using five different statistical algorithms. We conclude that *LsRPL5*, *Lsα-TUB*, *LsRPL8*, and *Lsβ-TUB* were the most suitable RT-qPCR reference genes for *L. striatellus* at different developmental stages, in different *L. striatellus* tissues, in RBSDV-V or RSV-V *L. striatellus*, and in RBSDV-V and *LsSYNJ1*-silenced *L. striatellus*. The results presented in this paper are useful for studies of gene function during *L. striatellus* development and in insect–virus interactions.

## Figures and Tables

**Table 1:** Primers of the candidate reference genes used in this study. Sequences of the primers are shown.

Gene Name	Gene Symbol	Gene ID	Primer Sequences (5′–3′)	Amplicon Length (bp)
actin	*ACT*	AY192151	*F*: TGAGCGTGAAATCGTAAGAGACAT	187
			*R*: GAAGGAAGGCTGGAATAGGG	
alpha-1-tubulin	*α-TUB*	AY550922	*F*: AGACAATGAGGCTATCTACGACA	296
			*R*: CCATCTGGTTGGCGGGTT	
beta-tubulin	*β-TUB*	AY334072	*F*: TACCGCCCATTGGTCTGC	167
			*R*: CGGCTTCAGTGAACTCCATCT	
Glyceraldehyde-3-phosphate dehydrogenase	*GAPDH*	HQ385974	*F*: ACGCACCCATGTTCGTGT	193
			*R*: CGGTCCGTCAACAGTCTTCT	
Ribosome protein L5	*RPL5*	HQ385973	*F*: CCGAAGTGACAGGCGAGGAG	164
			*R*: CACGCTGTGCGGGATGTT	
Ribosome protein L8	*RPL8*	HQ385976	*F*: AGGGAGCGGGAAGTGTTTT	267
			*R*: CCAATCTGTAGAGTGGCTTTC	
18s ribosome RNA	*18s rRNA*	AB085211	*F*: GTAACCCGCTGAACCTCC	169
			*R*: GTCCGAAGACCTCACTAAATCA	

**Table 2:** Expression stability ranking of the seven reference gene expression stabilities using the 2 ^−^^△△Ct^ method, geNorm, Bestkeeper, Normfinder, or the RefFinder algorithm.

Rank	Delta Ct			geNorm		Bestkeeper		Normfinder		RefFinder	
	Gene Name	Average Ct	SD	Gene Name	M	Gene Name	CV	Gene Name	SV	Gene Name	GM
1	*18S rRNA*	14.10	1.22	*α-TUB*	1.67	*18S rRNA*	1.17	*α-TUB*	1.34	*α-TUB*	1.41
2	*β-TUB*	24.07	1.62	*β-TUB*	1.67	*ACT*	1.28	*GAPDH*	1.45	*β-TUB*	2.28
3	*GAPDH*	19.66	1.81	*RPL5*	1.81	*β-TUB*	1.29	*β-TUB*	1.47	*GAPDH*	2.99
4	*α-TUB*	20.44	1.86	*GAPDH*	2.12	*α-TUB*	1.47	*RPL5*	1.71	*18S rRNA*	3.98
5	*ACT*	19.87	2.10	*RPL8*	2.19	*GAPDH*	1.50	*RPL8*	1.94	*RPL5*	4.28
6	*RPL5*	20.61	2.10	*18S rRNA*	2.32	*RPL8*	1.66	*ACT*	1.98	*ACT*	4.92
7	*RPL8*	20.46	2.22	*ACT*	2.39	*RPL5*	1.69	*18S rRNA*	1.99	*RPL8*	5.23

Note: Reverse transcription quantitative polymerase chain reaction (RT-qPCR) data were analyzed using different *Laodelphax striatellus* tissues, *L. striatellus* at different developmental stages, non-viruliferous (NV), *Rice black-streaked dwarf virus* viruliferous (RBSDV-V), *Rice stripe virus* viruliferous (RSV-V) *L. striatellus*, or RBSDV-V and Lssynaptojanin 1 (*LsSYNJ1*)-silenced *L. striatellus*. SD, standard deviations; M, globe gene expression stability values; CV: coefficients of variation; SV, stability values; GM, geometric mean.

## Supplementary Materials

The following are available online at https://www.mdpi.com/2073-4425/10/11/887/s1, Figure S1. Primer specificities of the selected candidate reference genes determined by RT-PCR and gel electrophoresis. Figure S2. Melting curves, melting peaks and amplification curves of the seven selected candidate reference genes. Figure S3. Amplification of RBSDV P10 and RSV CP in RBSDV-V and RSV-V sample by RT-PCR, respectively. Figure S4. The relative expression of *LsSYNJ1* in *Laodelphax striatellus* injected *dsGFP* and *dsSYNJ1*. Table S1.Primers used for qRT-PCR analysis of RBSDV, RSV gene expression and for dsRNA synthesis. Table S2. Expression stability ranking of the selected candidate reference genes using geNorm, Bestkeeper, Normfinder, and RefFinder.
