# Supplementary material for: Determination of Suitable RT-qPCR Reference Genes for Studies of Gene Functions in Laodelphax striatellus (Fallén)
Source: Genes (Basel). 2019 Nov 4;10(11):887. doi: 10.3390/genes10110887 (PMC6896117; doi:10.3390/genes10110887)
Supplement: Supplementary file 1 [file genes-10-00887-s001.zip › Supplementary files/Supplemental figure legend.docx]

Supplemental figure legend:

Figure S1. Primer specificities of the selected candidate reference genes determined by RT-PCR and gel electrophoresis.

Figure S2. Melting curves, melting peaks and amplification curves of the seven selected candidate reference genes.

Figure S3. Amplification of RBSDV *P10* and RSV *CP* in RBSDV-V and RSV-V sample by RT-PCR, respectively.

S4. The relative expression of *LsSYNJ1* in *Laodelphax striatellus* injected *dsGFP* and *dsSYNJ1*.
