# Supplementary figures and images for: Determination of Suitable RT-qPCR Reference Genes for Studies of Gene Functions in Laodelphax striatellus (Fallén)
Source: Genes (Basel). 2019 Nov 4;10(11):887. doi: 10.3390/genes10110887 (PMC6896117; doi:10.3390/genes10110887)

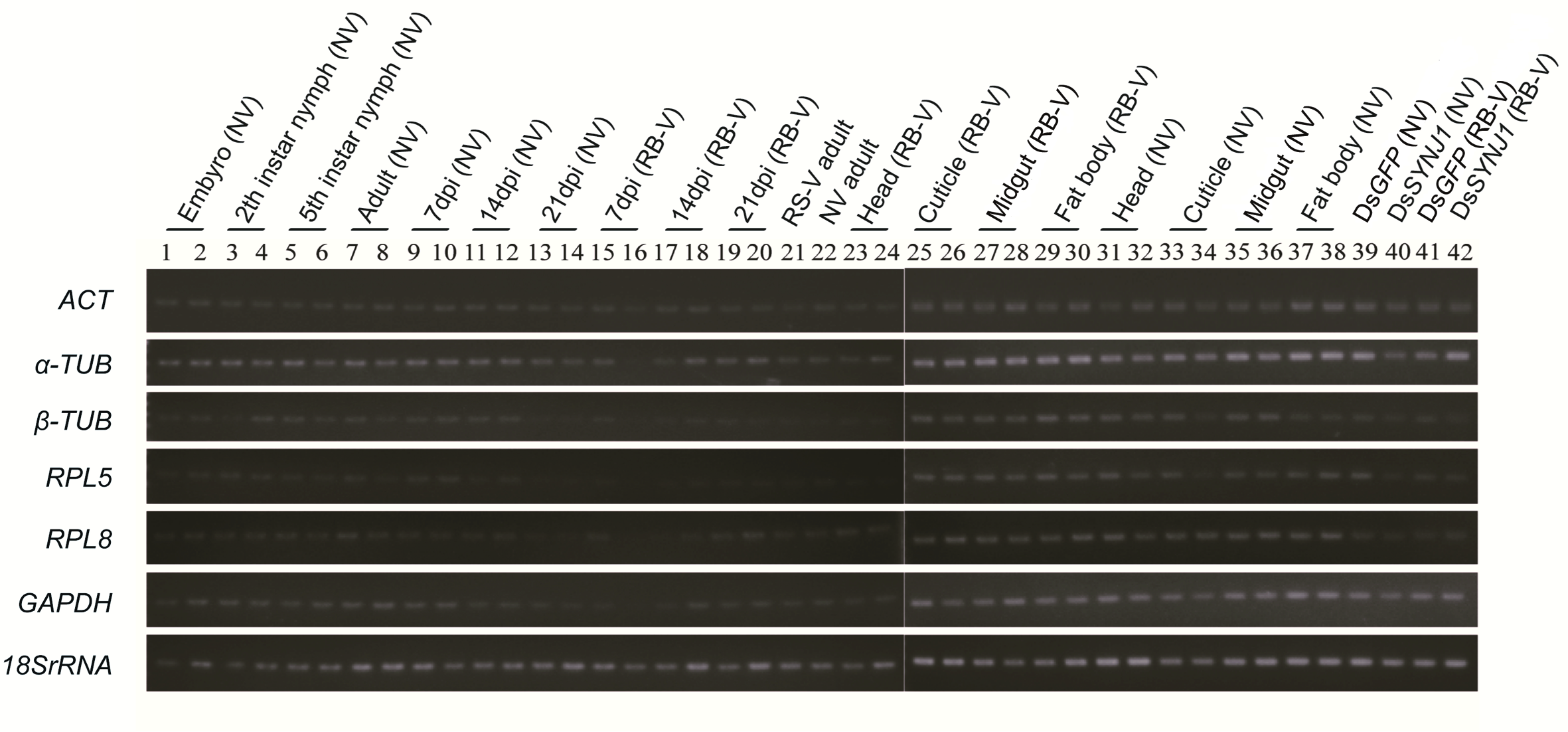

Supplement: Supplementary file 1 [file genes-10-00887-s001.zip › Supplementary files/Figure S1.tif]

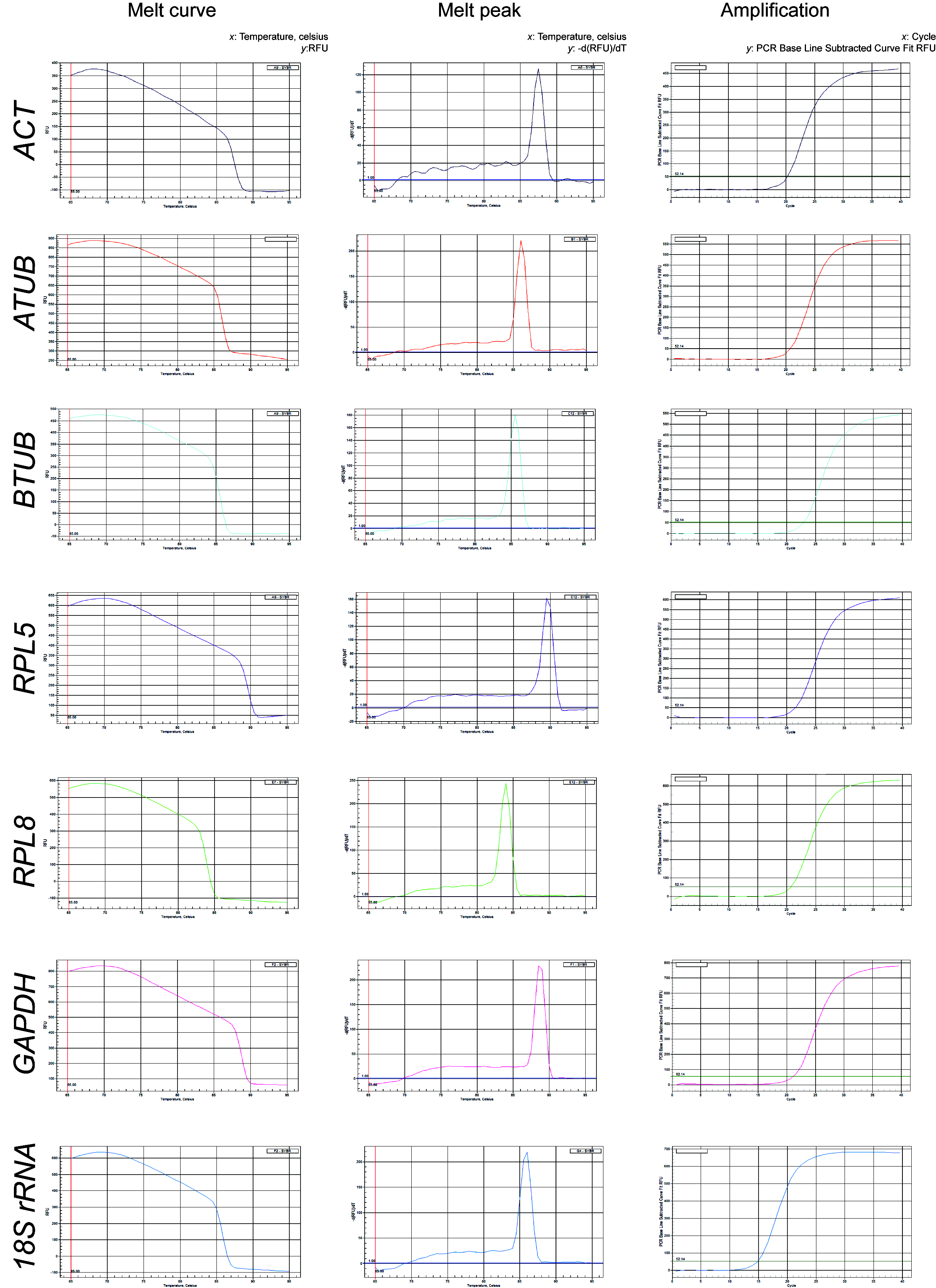

Supplement: Supplementary file 1 [file genes-10-00887-s001.zip › Supplementary files/Figure S2.tif]

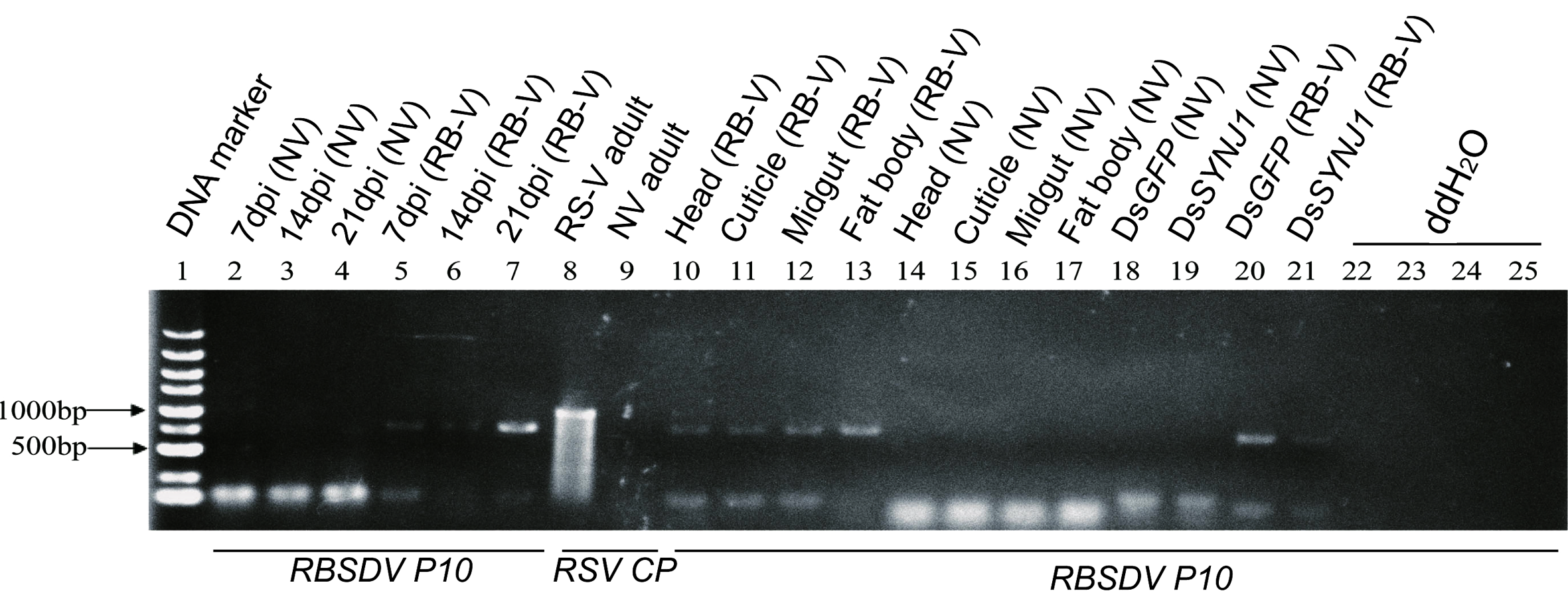

Supplement: Supplementary file 1 [file genes-10-00887-s001.zip › Supplementary files/Figure S3.tif]

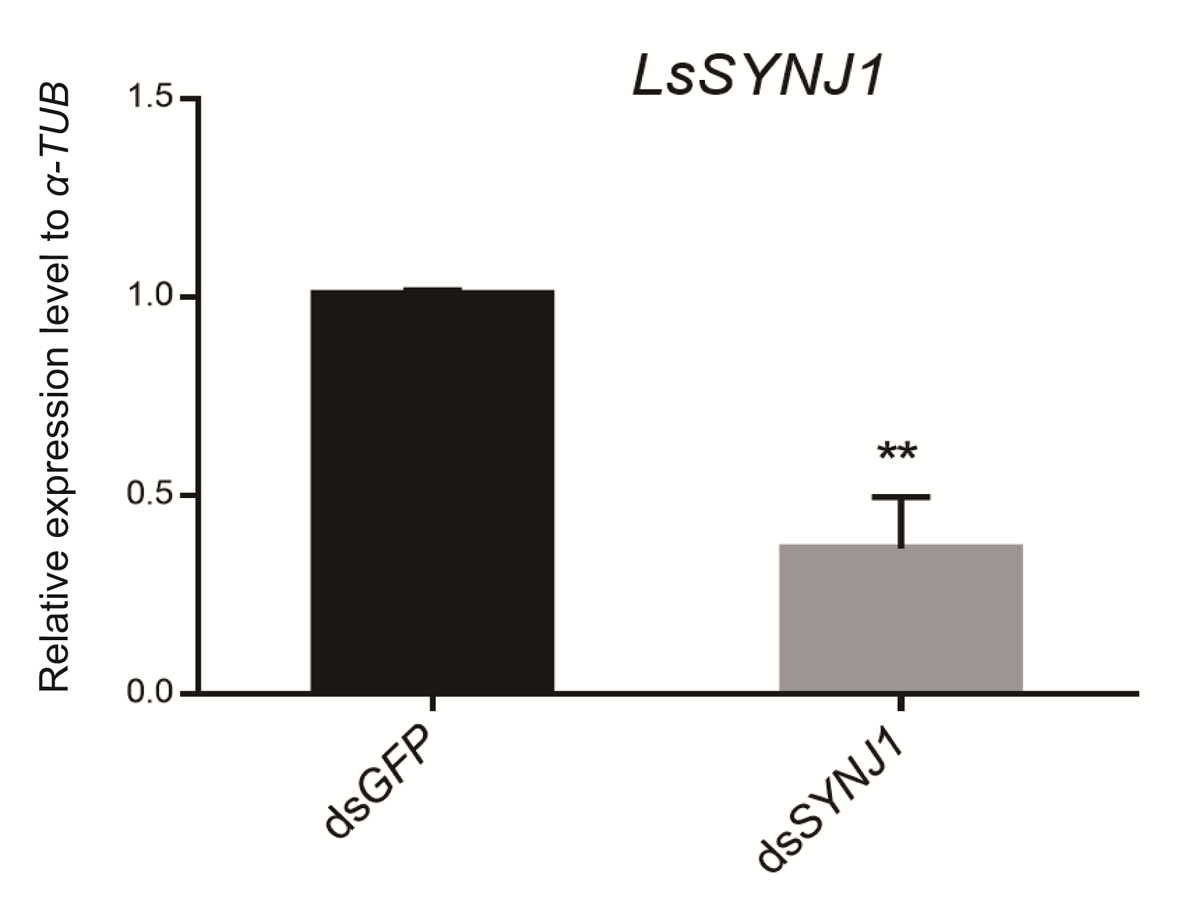

Supplement: Supplementary file 1 [file genes-10-00887-s001.zip › Supplementary files/Figure S4.tif]
